# Ethnic disparities in clozapine prescription for service-users with schizophrenia-spectrum disorders: a systematic review

**DOI:** 10.1017/S0033291722001878

**Published:** 2022-09

**Authors:** Anita Margarette Bayya Ventura, Richard D. Hayes, Daniela Fonseca de Freitas

**Affiliations:** 1 Department of Psychological Medicine, Institute of Psychiatry, Psychology & Neuroscience, King's College London, London, UK; 2 Department of Psychiatry, University of Oxford, Oxford, UK

**Keywords:** clozaril, disparities, healthcare, race, refractory psychosis, zaponex

## Abstract

Clozapine is the only licenced medication for treating treatment-resistant schizophrenia. Previous studies have suggested unequal rates of clozapine treatment by ethnicity among individuals with schizophrenia-spectrum disorders. One previous review has investigated this topic but was restricted to studies from the USA. This current review aims to synthesise the international literature regarding ethnic disparities in clozapine prescription amongst individuals with schizophrenia-spectrum disorders. We searched CINAHL, PubMed, Medline, Embase, APA PsycINFO and Open Grey and reviewed studies reporting on the proportion of service-users prescribed clozapine separately for different ethnic groups, in individuals with a primary diagnosis of schizophrenia or any schizophrenia-spectrum disorders. A narrative synthesis was conducted to integrate information from included studies. The review was registered in PROSPERO (Number: CRD42020221731). From 24 studies, there is strong, consistent evidence that Black and Hispanic service-users in the UK and the USA are significantly less likely to receive clozapine than White/Caucasian service-users after controlling for multiple demographic and clinical potential confounders. In New Zealand, Māori service-users were reported to be more likely to receive clozapine than those of White/European ethnicity. There is mixed evidence regarding Asian service-users in the UK. The mentioned disparities were observed in studies with TRS and non-TRS cohorts. The results imply that access to clozapine treatment varies among ethnic groups. These findings raise an ethical concern as they suggest a compromise of the standards of care in schizophrenia treatment practices. Interventions are needed to reduce clozapine prescribing disparities among ethnic communities.

## Introduction

Schizophrenia is a highly debilitating psychiatric disorder affecting up to 20 million people worldwide (James et al., 2018). About one-third of individuals with schizophrenia show inadequate response to consecutive treatments with two different antipsychotics, showing treatment-resistance (Farooq, Choudry, Cohen, Naeem, & Ayub, 2019; NICE, 2014). Currently, clozapine, a second-generation antipsychotic (SGA), is the only licenced medication for treating treatment-resistant schizophrenia (TRS) (Farooq et al., 2019).

Independently-funded randomised controlled trials and meta-analyses have shown that clozapine boasts superior efficacy compared to other SGAs, regarding amelioration of positive and extrapyramidal symptoms, in TRS patients (Chakos, Lieberman, Hoffman, Bradford, & Sheitman, 2001; Lewis et al., 2006). Another meta-analysis found significantly higher reductions in positive and negative symptoms in TRS patients being treated with clozapine than in TRS patients taking other first or SGAs (Siskind, McCartney, Goldschlager, & Kisely, 2016). The higher efficacy of clozapine in treatment-resistance may be due to being a strong 5-HT2A receptor antagonist and its power to mitigate the release of glutamate (Gillespie, Samanaite, Mill, Egerton, & MacCabe, 2017). Furthermore, there is evidence to suggest that clozapine is associated with reduced hospitalisation (Kesserwani et al., 2019), mortality (Vermeulen et al., 2019), and is a more cost-effective treatment for TRS, compared to the use of other antipsychotics (Jin, Tappenden, MacCabe, Robinson, & Byford, 2020). International clinical guidelines recommend using clozapine at the earliest opportunity once an individual has been identified with treatment-resistance (Lehman et al., 2004; NICE, 2014). Failure to prescribe clozapine is a major obstacle to optimal care for individuals with TRS and compromises the standard of care that healthcare providers are legally and ethically expected to uphold (Verdoux, Quiles, Bachmann, & Siskind, 2018; World Health Organisation, 2017).

Findings from previous studies suggest that access to clozapine may vary by ethnicity (Copeland, Zeber, Valenstein, & Blow, 2003; Das-Munshi, Bhugra, & Crawford, 2018; Kuno & Rothbard, 2002; Wheeler, Humberstone, & Robinson, 2008), where patients from minoritised ethnic backgrounds are less likely to be treated with clozapine compared to White/Caucasian service-users. The results from a recent systematic review of studies from the USA suggest that Black, African-American and Hispanic service-users are less likely to receive clozapine relative to White service-users (Williams, Harowitz, Glover, Tek, & Srihari, 2020). However, given that this previous investigation was limited to American-based studies, such findings may not be extrapolated to other countries. There is evidence of ethnic inequalities in New Zealand (Wheeler et al., 2008) and the UK (Das-Munshi et al., 2018), but it is currently unknown whether ethnic disparities in access to clozapine treatment are transculturally observed or are specific to some countries or ethnicities. This systematic review aims to address these gaps in the literature by synthesising the international literature regarding ethnic differences in clozapine prescription in cohorts of service-users with schizophrenia-spectrum disorders.

## Methods

### Protocol and registration

This systematic review was conducted following the reporting guidelines of the PRISMA-E statement (Welch et al., 2015) and two additional guidelines (Munn, Moola, Lisy, Riitano, & Tufanaru, 2020; Pai et al., 2004). The narrative synthesis was conducted following the guidelines presented by Popay et al. (2006). The protocol of this review was registered on PROSPERO (registration number CRD42020221731).

### Search strategy

The search strategy was developed by first conducting a scoping search of articles related to ethnic disparities in clozapine prescription. Titles and abstracts of these articles were read to generate keywords related to two concepts: ‘clozapine’ and ‘ethnicity’. Synonyms related to these concepts were further researched and generated. To capture the culturally-sensitive language used to describe ethnic groups across various countries, ethnicity-related keywords were generated by researching government-recognised ethnicity terminology, from official government websites across five countries: Australia (Australian Bureau of Statistics, 2019); Canada (Statistics Canada, 2016); New Zealand (Statistics New Zealand, 2005); the UK (UK Government, 2011) and the USA (United States Census Bureau, 2020). Both clozapine and ethnicity-related keywords were used to build a common free-text search using Boolean operators and truncators, which was used across all databases. Medical subject headings (MeSH) searches were conducted in each database alongside the free-text search, to capture relevant terminology not already included in the free-text search (full search strategy available in online Supplementary Material). The following six electronic databases were searched: CINAHL, PubMed, Medline, Embase, APA PsycINFO and Open Grey. Searches for each database were performed on 13 December 2020. The reference lists of included studies were searched for additional eligible studies. No time-period or language filters were applied during database searching.

Ethnicity is a multi-faceted social construct that encompasses the various ways individuals identify with a group of people based on shared experiences, cultural or religious affiliations, language, geographical and familial origins (Bhopal, 2004). The term ‘ethnic minority’ describes any ethnic group other than the culturally dominant ethnic group of the country in context. For example, for studies conducted in the UK, the culturally dominant group is White-British (UK Government, 2020); any ethnic group outside of White-British would be considered an ethnic minority. It is recognised that the terms ‘ethnicity’ and ‘race’ are often used interchangeably and synonymously throughout the literature (Bhopal, 2004), and is usually based on an individual's self-report or clinician's observations. For this review, the same ethnic terminology of the primary study has been used when referencing its findings.

### Study selection, data extraction, and quality assessment

For study selection, the retrieved records were screened against the following inclusion criteria, where studies were considered if they: (1) comprised samples of ⩾ 90% service-users with a primary diagnosis of schizophrenia or any schizophrenia-spectrum disorders, as diagnosed using official diagnostic manuals (e.g. The International Statistical Classification of Diseases and Related Health Problems (ICD, World Health Organisation, 2019) or Diagnostic and Statistical Manual of Mental Disorders (DSM, American Psychiatric Association, 2013)) and (2) reported the proportion of service-users prescribed clozapine separately for different ethnic groups. During each stage of study selection, data extraction and quality assessment, a randomly selected 10% of the records were screened independently by A.M.B.V and D.F.F and then A.M.B.V conducted the screening, data extraction and quality assessment of all remaining studies. The initial inter-rater agreement for the abstract screening was modest (Cohen's *k* = 0.45) and for the full-text screening was moderate (*k* = 0.72) (McHugh, 2012). A conservative approach was adopted during screening in which when there were doubts regarding inclusion a decision would be made involving all team members.

On the narrative synthesis, we looked for patterns in the odds ratio of clozapine treatment for each ethnic minority group as compared to the culturally dominant ethnic group across studies in that country (Popay et al., 2006). In doing this, we considered studies' sampling methods, adjustment for confounders and considered study quality; giving greater weight to the evidence provided by high-quality studies. We have also investigated if potential disparities were moderated by TRS, i.e. if disparities were only observed in cohorts of people with, or without, treatment-resistance. The extracted data included information regarding study design, sample characteristics, the percentages of clozapine prescription for each ethnic group and the odds ratio for treatment with clozapine for each ethnic group. When no statistical analyses were published in the original studies, the odds ratio and respective 95% confidence intervals were calculated using published formulas (Tenny & Hoffman, 2021). The data extraction form is available in the online Supplementary Material. The quality of the information retrieved in each study was assessed based on 11 items adapted from the Checklists for Prevalence Studies and Cross-Sectional Studies from the Johanna Briggs Institute (Joanna Briggs Institute, 2020). Changes to the original questions were made to reflect better the nature of the research question and any potential biases related to the quality of information retrieved regarding ethnicity and clozapine (assessment checklist and results for quality assessment is available in online Supplementary Material). Studies were sorted into one of three categories based on the quality of information: high (10–11 points), medium (8–9 points), or low (0–7 points).

## Results

### Study selection characteristics

**Fig. 1.**
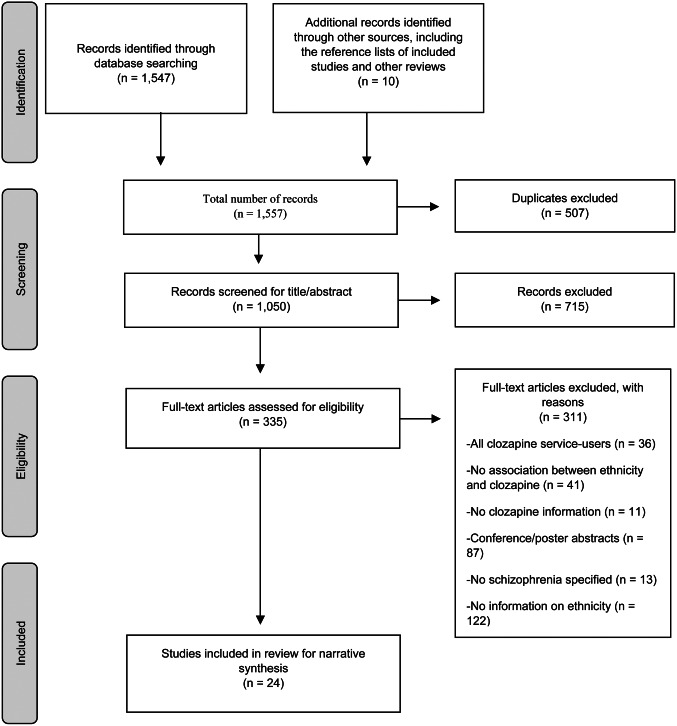
Flow chart summarising the literature search and study selection process [adapted from Moher, Liberati, Tetzlaff, and Altman (2009)].

A total of 1557 potentially relevant records were identified, and duplications were removed, leaving 1050 records. These records were screened and twenty-four studies met the eligibility criteria. Figure 1 summarises the results of the literature search and screening process. The study characteristics are summarised in Table 1. The 24 studies were conducted in four countries: Brazil (*k* = 1), New Zealand (*k* = 3), the UK (*k* = 6) and the USA (*k* = 14). There was a wide range of ethnic groups investigated, including but not limited to African-American and Hispanic in the USA; Asian/Asian-British and Black/Black-British in the UK; Māori and Pacific Islander in New Zealand, and Caucasian/European/White (in all countries). The culturally dominant ethnic group, for all countries in the context of this review, was Caucasian/European/White.

**Table 1.** Summary of study characteristics and results

Author (Year), Country	Primary study focus	Data source, Observation period	Inclusion criteria, TRS working definition (if applicable); Total Sample (*N*)	Proportion of clozapine prescription and statistical tests^a^
Brazil				
de Almeida et al. (2020)	Health-related quality of life and associated factors	*Data:* Participants recruited from a Specialised Pharmaceutical Service in Belo Horizonte; data extracted from self-reported questionnaire *Period:* 09/2017–03/2018	*Inclusion Criteria:* Individuals taking one antipsychotic and who had completed the quality-of-life questionnaire ***N*** = 394	*Caucasian/Asian:* 14.8% *Mixed/Afro-Brazilian:* 18.4%; OR 1.30, 95% CI [0.75–2.26] *Unknown ethnicity:* 6.7%; OR not calculated due to small sample size
New Zealand				
Dey et al. (2016)	Clinical compliance with prescribing guidelines and clinicians' characteristics	*Data:* Medical records from three inpatient units from the regions of Waikato, Tairawhiti and Lakes *Period:* 07/2009–12/2011	*Inclusion criteria:* Inpatients with an ICD-10 diagnosis of schizophrenia or related disorders ***N*** = 451	*Non-Māori:* 13% *Māori:* 24%; OR 2.10, 95% CI [1.26–3.50]
Wheeler et al. (2008)	Antipsychotic prescription patterns between ethnic groups	*Data:* Clinical files from community mental health services in Auckland *Period:* **T1**: 03/2000, **T2**: 10/2004	*Inclusion Criteria:* Outpatients with a DSM-IV diagnosis of schizophrenia or schizoaffective disorder ***N*** = 2236 (T1), 2506 (T2)	*European* **T1**: 22.9%, **T2**: 34.4% *Māori* **T1**: 19.2%; OR 0.80, 95% CI [0.62–1.05], **T2**: 38.8%; OR 1.21, 95% CI [0.99–1.48] *Pacific-Islander* **T1**: 19.1%; OR 0.79, 95% CI [0.59–1.07], **T2**: 32.9%; OR 0.93, 95% CI [0.75–1.17] *Asian* **T1**: 11.4%; OR 0.43, 95% CI [0.25–0.73], **T2**: 20.2%; OR 0.49, 95% CI [0.32–0.74] Adjusted for age and gender: **T1**: *p* *=* 0.007, **T2**: *p* *<* 0.001
Wheeler (2008)	Patterns of clozapine usage	*Data:* Clinical files from Community Mental Health Services and District Health Board patient's information management system *Period:* 10/2004	*Inclusion Criteria:* Outpatients aged between 15-64 years of age, with a DSM-IV diagnosis of schizophrenia or schizoaffective disorder who were prescribed an antipsychotic ***N*** = 2796	*European:* 32.0% *Māori:* 36.1%; OR 1.20, 95% CI [1.00 −1.45] *Pacific Nations:* 34.5%; OR 1.12, 95% CI [0.90–1.39] *Asian:* 20.3%; OR 0.54, 95% CI [0.35–0.83] *Other:* 19.4%; OR 0.51, 95% CI [0.21–1.25]
United Kingdom				
Beck et al. (2019)	Prevalence of TRS and clozapine use	*Data:* Case notes from two Community Mental Health Teams, part of SLAM services *Period:* 09/2014–09/2015	*Inclusion Criteria:* Individuals with an ICD-10 SSD diagnosis; *N* = 176 *TRS Definition:* Presenting persistent psychotic symptoms, functional impairment and at least two adequate trials with different antipsychotics; ***N*** = 99	*White:* 69% *Non-White:* 40%; OR 0.30, 95% CI [0.12–0.75]
Cho et al. (2019)	Clozapine treatment and all-cause mortality within a TRS cohort	*Data:* Electronic health records from SLAM services *Period:* 01/2008–12/2016	*Inclusion criteria:* Service-users who were 15 years of age or older, with an ICD-10 SSD diagnosis and who received treatment from SLAM services between 01/2007–01/2016 and had TRS *TRS definition:* Having three treatments with three different antipsychotics where the third antipsychotic was initiated during hospitalisation; ***N*** = 2837	*White:* 45.5% *Other/Not Stated:* 40.0%; OR not calculated due to small sample size *Asian:* 38.6%; OR 0.75, 95% CI [0.54–1.06] *Mixed:* 35.4%; OR 0.66, 95% CI [0.49–0.88] *Black:* 29.4%; OR 0.50, 95% CI [0.42–0.59]
Das-Munshi et al. (2018)	Ethnic differences in mental health care treatments	*Data:* National Audit of schizophrenia (survey with clinicians) and Clinical Records from Mental Healthcare Trusts and Health Boards across England and Wales *Period:* 2011–2013	*Inclusion criteria:* Service-users over 18 years of age, with an ICD-10 SSD diagnosis before 60 years of age, who are under the care of community-based services; ***N* *=* *10 504*** *TRS definition:* Clinicians' assessment of significant disability or no remission; ***N*** *=* 2793	*White:* 26.8% *Mixed:* 28.3%; OR 1.08, 95% CI [0.59–1.98] *Chinese/Other:* 26.1%; OR 0.97, 95% CI [0.50–1.88] *Asian/Asian-British:* 23.4%; OR 0.83, 95% CI [0.61–1.14] *Black/Black-British:* 18.4%; OR 0.62, 95% CI [0.44–0.86]
Govind et al. (2020)	Risk of COVID-19 infection and clozapine treatment	*Data:* Electronic health records from SLAM services *Period* for antipsychotic use: 12/2019–03/2020	*Inclusion criteria:* Individuals receiving outpatient or inpatient care using SLAM services, with an ICD-10 SSD diagnosis, taking antipsychotic medication ***N*** *=* 6309	*White:* 24.6% *Asian:* 19.7%; OR 0.75, 95% CI [0.62–0.91] *Black:* 18.4%; OR 0.69, 95% CI [0.60–0.79]
Kesserwani et al. (2019)	Clozapine use and hospital readmission	*Data:* Electronic health records from SLAM services *Period:* 01/2007–12/2014	*Inclusion criteria:* Individuals aged between 15-95 years of age with an ICD-10 SSD diagnosis, with at least one inpatient episode where they were discharged on an antipsychotic ***N*** = 3651	*White:* 7.4% *South Asian:* 11.3%; OR 1.60, 95% CI [0.74–3.46] *Other Black:* 5.3%; OR 0.71, 95% CI [0.50–0.99] *Mixed-Unknown:* 5.2%; OR 0.69, 95% CI [0.39–1.22] *Other White:* 5.1%; OR 0.67, 95% CI [0.37–1.21] *East Asian:* 4.3%; 0.57, 95% CI [0.24–1.33] *Black Caribbean:* 2.5%; OR 0.32, 95% CI [0.17–0.60]
Stokes et al. (2020)	Prevalence of TRS and the pathways to clozapine initiation	*Data:* Participant data from the National Evaluation of the Development and Impact of Early Intervention Services Study in England *Period:* 04/2009–04/2010	*Inclusion criteria:* Individuals between 14-35 years of age, with an ICD-10 diagnosis of first-episode psychosis who were enrolled into early intervention services; *N* = 1027 *TRS definition:* Persistent psychotic symptoms (PANSS score of ⩾16, equating to at least two positive items of at least moderate severity) and at least two trials of different antipsychotics ***N*** = 81	*White:* 23.7% *Asian:* 57.1% *Black:* 50.0% *Mixed:* 33.3% *Other:* 0.0% ORs not calculated due to small sample size
United States of America				
Copeland et al. (2003)	Antipsychotic prescription patterns by ethnicity amongst a veteran sample	*Data:* Outpatient pharmacy and administrative records from the National Veterans Administration Psychoses Registries *Period:* 10/1998–09/1999	*Inclusion criteria:* Veterans with a diagnosis of schizophrenia and who were prescribed antipsychotic medications as outpatients ***N*** = 69 787	*White:* 3.6% *African-American:* 1.4%; OR 0.35, 95% CI [0.31–0.39] *Hispanic:* 1.2%; OR 0.33, 95% CI [0.26–0.41] *ORs adjusted for:* Age, gender, and other psychiatric diagnoses (substance use disorder, bipolar and other psychosis)
Horvitz-Lennon et al. (2013)	Effectiveness of clozapine by ethnicity relative to other antipsychotics	*Data:* Pharmacy and medical claims from Florida Medicaid Program *Period:* 07/2000–06/2005	*Inclusion criteria:* Individuals aged between 18-64 years of age with an ICD-9 diagnosis of schizophrenia and with at least one antipsychotic prescription ***N* = 20 122**	*White:* 5.9% *Black:* 2.3%; OR 0.37, 95% CI [0.30–0.46] *Latino:* 2.1%; OR 0.33, 95% CI [0.28–0.40]
Horvitz-Lennon et al. (2014)	Ethnic disparities in the quality of treatment for schizophrenia	*Data:* Medicaid claims data from California, New York, Florida, North Carolina *Period*: 2002–2009	*Inclusion criteria:* Individuals between 18-64 years of age, who were continuously enrolled in fee-for-service programmes, with ⩾2 outpatient or ⩾1 inpatient claims with an ICD-9 diagnosis of schizophrenia. Individuals could contribute multiple treatment-episodes Number of person-episodes: California = 139 065, Florida = 25 146, New-York = 120 704, North Carolina = 40 458	*White:* California – 9.2%; Florida – 8.6%; New York – 12.0%; North Carolina – 8.3% *Latino:* California – 6.8%, OR 0.45, 95% CI [0.42–0.47]; Florida – 3.3%; OR 0.36, 95% CI [0.31–0.42]; New York – 4.8%, OR 0.37, 95% CI [0.35–0.39]; North Carolina – 13.0%; OR 1.65, 95% CI [1.26–2.16] *Black:* California – 4.0%, OR 0.41, 95% CI [0.39–0.44]; Florida – 2.8%, %, OR 0.31, 95% CI [0.26–0.36]; New York – 4.7%, OR 0.36, 95% CI [0.34–0.38]; North Carolina – 4.2%, OR 0.48, 95% CI [0.44–0.53]
Kelly et al. (2006)	Ethnic differences in clozapine prescription and treatment outcomes	*Data:* Records from The Clozapine Authorization and Monitoring Program, The State of Maryland Antipsychotic Database and The Health Maintenance Information System Database *Period:* 03/1994–12/2000	*Inclusion criteria:* Individuals with a DSM-IV diagnosis of schizophrenia or schizoaffective disorder ***N*** = 2911	*White:* 15.3% *African-American:* 10.3%; OR 0.63, 95% CI [0.51–0.79]
Kelly et al. (2010)	Clozapine usage and cardiovascular risk	*Data:* Administrative data recording inpatient use of SGAs and records from the Clozapine Authorisation and Monitoring System *Period:* 01/1994–05/2000	*Inclusion criteria:* Individuals between 20-69 years of age, taking either clozapine or risperidone and with either a DSM-III/IV diagnosis of schizophrenia or schizoaffective disorder ***N*** = 1686	*White:* 70.3% *Other:* 73.6%; OR 1.18, 95% CI [0.65–2.17] *African-American:* 54.6%; OR 0.51, 95% CI [0.41–0.62]
Kreyenbuhl et al. (2003)	Ethnic differences in prescription rates	*Data:* Medical records from inpatient and outpatient treatment facilities in Maryland *Period:* 12/1994–03/1996	*Inclusion criteria:* English-speaking individuals, aged 18 years or older, with a diagnosis of schizophrenia or schizoaffective disorder and living within the community ***N*** = 344	*Caucasian:* 19.0% *African-American:* 3.0%; OR 0.13, 95% CI [0.05–0.37]
Kuno and Rothbard (2002)	Ethnic variations in the prescriptions of different antipsychotics	*Data Source:* Medicaid mental health claims records *Period:* 1995	*Inclusion criteria:* Medicaid recipients between 18-64 years of age, who were treated for schizophrenia with an antipsychotic ***N*** *=* 2515	*Caucasian:* 15.0%; OR 1.66, 95% CI [1.26–2.18]; OR Reference: African-American *African-American:* 8.2% *ORs adjusted for:* Gender, age, supplemental security income (SSI), service use (including psychiatric hospitalisation, emergency room visit, intensive case management, partial hospitalisation, medical care, drug, or alcohol treatment), and treatment with depot antipsychotic
Mallinger et al. (2006)	Ethnic differences in SGA use in outpatients	*Data:* Administrative and pharmacy data associated with an academic centre in New York *Period:* 2003–2004	*Inclusion criteria:* Individuals older than 18 years of age with a DSM-IV diagnosis of schizophrenia or schizoaffective disorder ***N*** = 456	*Caucasian:* 26.4%; OR 2.70, 95% CI [1.58–4.63]; OR Reference: African-American *African-American:* 12.2% *ORs adjusted for:* Age, gender, main diagnosis, insurance, education, living situation, substance abuse disorder
Manuel et al. (2012)	Demographic and clinical factors associated with the use of several antipsychotics	*Data:* New York State Medicaid claims records *Period:* 2008–2009	*Inclusion Criteria:* Individuals with an ICD-9 SSD diagnosis with at least one clinic service and an antipsychotic prescription, and continuous Medicaid eligibility throughout the observation period ***N*** *=* 7035	*Caucasian:* 2.8% *Black:* 1.6%; OR 0.57, 95% CI [0.38–0.86]; *Hispanic:* 1.2%; OR 0.47, 95% CI [0.24–0.91] *Other:* 2.2%; OR not provided by authors *ORs adjusted for:* Age, gender, type of facility where the claim was filed, the number of prescriptions filled for different antipsychotics, substance-abuse diagnosis, number of patient admissions, total Medicaid psychiatric costs
Mark et al. (2003)	Examining ethnic differences in treatment, symptom presentation and service use	*Data:* Medical records from Community Mental-Health services, Veterans Administration Systems, Inpatient settings, and State Hospitals in North Carolina, Connecticut, Maryland, California, Colorado, and Florida *Period:* 07/1999–07/2001	*Inclusion Criteria:* Individuals who are older than 18 years of age and with a DSM-IV diagnosis of schizophrenia or schizoaffective or schizophrenia-form disorder ***N*** = 2239	*Non-Black:* 16.9% *Black:* 5.5%; OR 0.29, 95% CI [0.21–0.40]
Ren et al. (2002)	Antipsychotic prescribing and related demographic and clinical characteristics	*Data:* Administrative Data from the 1999 National Health Survey of Veterans Administration (VA) Enrolees, VA national administrative records and VA National Pharmacy Benefits Management Program *Period:* 07/1998–06/1999	*Inclusion Criteria:* Individuals with at least one inpatient or ⩾2 outpatient events a diagnosis of an ICD-9 SSD disorder and who were at least on one antipsychotic medication ***N* = 74 715**	*White:* 2.6% *Black:* 0.9%; OR 0.36, 95% CI [0.31–0.43] *Hispanic:* 0.9%; OR 0.35, 95% CI [0.27–0.48] *Other:* 0.8%; OR 0.29, 95% CI [0.22–0.37]
Rothbard et al. (2003)	Patterns in antipsychotic use	*Data:* Enrolment files and paid claims records for services and pharmacy use for Medicaid-enrolled Philadelphia Residents *Period:* 1995	*Inclusion Criteria:* Individuals enrolled onto a Medicaid fee-for-service programme, between 18-64 years of age, with at least one ambulatory visit with an ICD-9 diagnosis of SSD ***N* = 2951**	*Caucasian:* 14.0% *African-American:* 7.9%; OR 0.53, 95% CI [0.41–0.68] *Other:* 7.8%; OR 0.52, 95% CI [0.34–0.80]
Stroup et al. (2014)	Predictors of clozapine use	*Data:* U.S Medicaid claims records from 45 states, supplemented by county-level information from Area Resource Files *Period:* 01/2002–12/2005	*Inclusion Criteria:* Medicaid-insured individuals between 18-64 years of age with an ICD-10 diagnosis of SSD and ⩾1 inpatient or ⩾2 outpatient claims, who were prescribed an antipsychotic in ⩾1 treatment-episodes. Individuals could contribute multiple treatment-episodes ***N* *=* *326 119*** (number of individuals); 629 809 (number of treatment-episodes) *TRS definition:* Prescription claims for >2 different antipsychotics with adequate medication adherence and >1 psychiatric hospitalisation ***N* *=* *79 934*** (number of treatment-episodes)	*White:* 3.0% *Other:* 2.30%; OR 0.89, 95% CI [0.84–0.94] *Hispanic:* 1.83%; OR 0.79, 95% CI [0.71–0.87] *African-American:* 1.68%; OR 0.66, 95% CI [0.61–0.72] *OR adjusted for:* TRS, gender, age, clinical comorbidities (including substance-abuse disorder, depression, anxiety, self-harm, diabetes, cardiovascular disease, HIV), schizophrenia sub-type, use of acute services (including mental-health emergency services, outpatient visits), hospital admissions, State (to control for in-state Medicaid programmes). Also, the authors report that when the analysis was restricted to the TRS cohort ORs were still significant but with attenuated magnitude.
Velligan et al. (2014)	Cost, usage, and outcomes of clozapine monotherapy or antipsychotic polypharmacy	*Data:* Administrative and pharmacy data from the Medicaid Market Scan Database *Period:* 07/2006–01/2009	*Inclusion Criteria:* Individuals with a DSM-9 diagnosis of schizophrenia and with at least one pharmacy claim for an SGA receiving clozapine monotherapy or antipsychotic polypharmacy ***N*** = 2919	*White:* 14.3% *Other:* 33.9%; OR 3.07, 95% CI [2.31–4.09] *Black:* 15.0%; OR 1.06, 95% CI [0.84–1.33]

*Notes:* Abbreviations: CI, Confidence Interval; DSM, Diagnostic and Statistical Manual of Mental Disorders (3rd or 4th Edition); ICD, The International Statistical Classification of Diseases and Related Health Problems (9th or 10th Edition); OR, Odds Ratio; RR, Relative Risk; SGAs, Second-Generation Antipsychotics; SLAM, South London and Maudsley NHS Foundation Trust; SSD, Schizophrenia-Spectrum Disorder; TRS, Treatment-Resistant Schizophrenia.

a Information regarding statistical tests is provided as presented in the original paper. When no statistical analyses were published in the original studies, the odds ratio and respective 95% confidence intervals were calculated, using published formulas (Tenny & Hoffman, 2021). The relative risk ratio (ORs/RRs) reported uses White/Caucasian service-users as the reference group unless otherwise stated.

Observation periods of the studies ranged from 1991 to 2020. Most studies employed retrospective cohort or cross-sectional study designs with a variety of data sources: electronic health records/clinical or administrative records (*k* = 7), medical claims/pharmacy data (*k* = 5), surveys/questionnaires (*k* = 2), or a combination of multiple data sources (*k* = 8). For two studies (Dey, Menkes, Obertova, Chaudhuri, & Mellsop, 2016; Stokes et al., 2020), the source of data was unclear. There was a large variation in the sample sizes of the included studies, which ranged from 202 to 326 119. Of 24 studies, only 12 aimed to investigate antipsychotic prescription practices by ethnicity. Out of 24 studies, seven reported the results of statistical tests between ethnicity and clozapine prescription. For the remaining 16 studies, the odds ratio were calculated. For one study (Stokes et al., 2020), odds ratios were not calculated due to the small sample size (see Table 1). Ten studies performed analyses that adjusted for either demographic (e.g. age, gender) or clinical covariates (e.g. substance-abuse comorbidity, service-use, TRS) or both. Five studies had restricted analysis to TRS samples only (Beck et al., 2019; Cho et al., 2019; Das-Munshi et al., 2018; Stokes et al., 2020; Stroup, Gerhard, Crystal, Huang, & Olfson, 2014).

The quality of information relevant to the research question of this review was categorised as high-quality in four studies, categorised as medium-quality in five studies, and low-quality in 15 studies (see online Supplementary Material, Table S10). Information regarded as low-quality usually was retrieved from studies whose original aim was not investigating associations between ethnicity and clozapine prescription.

### Clozapine prescription of minority ethnic service-users compared to White/Caucasian service-users

#### Studies from the Americas

Most studies conducted in the USA (13 of 14) report ethnic inequalities in treatment with clozapine (Table 1). Several high and medium-quality studies showed that African-American service-users had between one to two thirds the odds of being treated with clozapine compared to White/Caucasian service-users, after controlling for several demographic and clinical covariates (Copeland et al., 2003; Kuno & Rothbard, 2002; Manuel, Essock, Wu, Pangilinan, & Stroup, 2012; Rothbard, Kuno, & Foley, 2003; Stroup et al., 2014). For example, Kuno and Rothbard (2002) had reported disparities after controlling for income, psychiatric hospitalisation, drug/alcohol treatment and intensive case management. A decade later, Manuel et al. (2012) also reported a disparity for African-American service-users after adjusting for substance abuse comorbidity, the number of patient admissions and total Medicaid psychiatric costs. More recently, Stroup et al. (2014) reported African-American service-users with TRS had 66% odds to receive clozapine compared to White service-users, after adjusting for diabetes and cardiovascular disease (CVD). This was the only study to adjust for these comorbidities, which could prevent treatment with clozapine. Some studies considered low-quality reported similar results (Kelly et al., 2006; Mallinger, Fisher, Brown, & Lamberti, 2006). Only one low-quality study from the USA reported no significant difference in the odds of receiving treatment between White and Black groups (Velligan, Carroll, Lage, & Fairman, 2014).

All but one study showed Hispanic and Latino service-users in the USA were significantly less likely to receive clozapine than White/Caucasian service-users after controlling for several demographic and clinical covariates (Copeland et al., 2003; Horvitz-Lennon, Donohue, Lave, Alegría, & Normand, 2013; Horvitz-Lennon et al., 2014; Manuel et al., 2012; Ren et al., 2002; Stroup et al., 2014). The only exception to this was reported in one low-quality study covering four states across the USA (Horvitz-Lennon et al., 2014); it was observed that in North Carolina Latino service-users were significantly more likely to receive clozapine, however, were less likely to receive clozapine in California, Florida and New York (Table 1). The strength of the association varied between studies. For example, after controlling for covariates, such as comorbid bipolar or substance abuse disorder, Copeland et al. (2003) reported that Hispanic service-users have only 33% odds of receiving clozapine, while Stroup et al. (2014) reported 79% odds after adjusting for diabetes, CVD and TRS.

Only one study from the USA controlled for TRS in analysis (Stroup et al., 2014). TRS was associated with almost two-fold the odds of being treated with clozapine (adjusted OR 1.92, 95% CI 1.83–2.03; Stroup et al., 2014). The authors report that when restricting analyses to the cohort of people with TRS, ethnic disparities (mentioned above) were still found to persist, but the magnitude was slightly reduced (odds ratio not provided). Considering the number of confounders included in this study (see Table 1 for full list) and the fact that it uses national data, the evidence suggests that is it not the incidence of TRS that is driving ethnic disparities in clozapine.

In South America, one study from Brazil (de Almeida et al., 2020), whose information was considered low-quality, found no significant differences in the rates of Afro-Brazilian or mixed heritage service-users in receiving clozapine treatment compared to Caucasian and Asian people (Table 1).

#### Studies from the United Kingdom

Studies conducted in the UK show that Black service-users are less likely to be treated with clozapine than White-British service-users (Beck et al., 2019; Cho et al., 2019; Das-Munshi et al., 2018; Govind, Fonseca de Freitas, Pritchard, Hayes, & MacCabe, 2020; Kesserwani et al., 2019). Das-Munshi et al. (2018), whose study used nationally representative data and whose information were considered as high-quality, reported that Black/Black-British service-users with TRS had only 62% the odds to receive clozapine than White service-users. Other studies (Cho et al., 2019; Govind et al., 2020; Kesserwani et al., 2019), whose information was from clinical healthcare records in South London, also reported significantly lower odds of clozapine treatment among Black service-users, compared to White service-users.

Studies reported mixed evidence for Asian service-users in the UK. Some studies reported no significant difference in the rates of Asian service-users receiving clozapine compared to White service-users. For example, a high-quality study with national data (Das-Munshi et al., 2018), found no significant difference in the likelihood of Chinese and Asian/Asian-British service-users with TRS receiving clozapine compared to White service-users. One study (Kesserwani et al., 2019), whose information was considered as low-quality, reported no significant difference in the likelihood of either South or East-Asians in receiving clozapine compared to White service-users. However, studies using healthcare record data from South London (Cho et al., 2019; Govind et al., 2020), suggests that Asian service-users have only 75% odds of receiving clozapine compared to White service-users.

Four of the six UK studies had samples of people with TRS (Beck et al., 2019; Cho et al., 2019; Das-Munshi et al., 2018; Stokes et al., 2020). Relatively similar findings were observed in the magnitude of disparities between Black and White-British people among studies with TRS samples (Beck et al., 2019; Cho et al., 2019; Das-Munshi et al., 2018) and those with non-TRS samples (Govind et al., 2020; Kesserwani et al., 2019). Mixed-evidence regarding disparities in treatment with clozapine between South Asian and White British were observed in studies with TRS (Cho et al., 2019; Das-Munshi et al., 2018) and non-TRS samples (Govind et al., 2020; Kesserwani et al., 2019). Thus, the current evidence does not suggest that the incidence of TRS is the reason for ethnic disparities in clozapine treatment in the UK.

#### Studies from New Zealand

In New Zealand, Asian service-users have significantly lower odds of receiving clozapine treatment compared to service-users of European ancestry, as reported by two medium-quality studies (Wheeler, 2008; Wheeler et al., 2008). For Pacific Islander service-users, there was no significant difference in the likelihood of clozapine treatment compared to European service-users reported (Wheeler, 2008; Wheeler et al., 2008). Regarding Māori service-users, three medium-quality studies (Dey et al., 2016; Wheeler, 2008; Wheeler et al., 2008) reported they were more likely to receive clozapine compared to European service-users. However, there is evidence to suggest that higher rates of treatment with clozapine among Māori service-users have only taken place in the last two decades. Wheeler et al. (2008) observed that in the year 2000, Māori service-users had lower treatment rates than Europeans, but in 2004, Māori were more likely to be prescribed clozapine compared to European service-users.

## Discussion

This systematic review aimed to synthesise the available evidence regarding possible ethnic disparities in clozapine treatment. Twenty-four studies, from four countries (Brazil, New Zealand, the UK, and the USA), were analysed. Black and Hispanic service-users in the UK and the USA were found to be consistently and significantly less likely to receive clozapine relative to White/Caucasian service-users (Beck et al., 2019; Cho et al., 2019; Copeland et al., 2003; Das-Munshi et al., 2018; Govind et al., 2020; Horvitz-Lennon et al., 2013, 2014; Kelly et al., 2006; Kesserwani et al., 2019; Kreyenbuhl, Zito, Buchanan, Soeken, & Lehman, 2003; Kuno & Rothbard, 2002; Mallinger et al., 2006; Manuel et al., 2012; Ren et al., 2002; Rothbard et al., 2003; Stroup et al., 2014; Velligan et al., 2014). This disparity was present in studies that controlled for various demographic and clinical covariates including treatment-resistance. In the only study included in the review from Brazil, no significant differences were reported in the rates of receiving clozapine for service-users of mixed race or Afro-Brazilian descent compared to those of Asian or Caucasian backgrounds (de Almeida et al., 2020). Mixed findings were observed regarding clozapine treatment within Asian communities in the UK (Cho et al., 2019; Das-Munshi et al., 2018; Govind et al., 2020; Kesserwani et al., 2019). In New Zealand, compared to European service-users, Asian service-users had significantly lower odds of clozapine prescription, but no differences were reported among Pacific-Islander service-users (Wheeler, 2008; Wheeler et al., 2008). Also, Māori service-users were more likely to receive clozapine than service-users of European origin (Dey et al., 2016; Wheeler, 2008; Wheeler et al., 2008). The findings of this review also suggest that the aforementioned ethnic inequities in clozapine treatment are observed in both treatment-resistant and non-TRS cohorts.

Several included studies have suggested that a potential reason for disparities in the use of clozapine in Black and African-American communities, is the presence of benign ethnic neutropenia (BEN) (Beck et al., 2019; Cho et al., 2019; Copeland et al., 2003; Das-Munshi et al., 2018; Kelly et al., 2006; Kreyenbuhl et al., 2003; Kuno & Rothbard, 2002; Mallinger et al., 2006; Manuel et al., 2012; Stroup et al., 2014). This is a hereditary condition seen in 25–50% of individuals of African and Middle Eastern descent and is defined as an absolute neutrophil count within the range of 1000–1800/mm^3^ (Manu, Sarvaiya, Rogozea, Kane, & Correll, 2016; Rajagopal, 2005). Current clinical guidelines in the USA recommend clozapine initiation be limited to those with an absolute neutrophil count of at least 2000/mm^3^, due to increased risk for clozapine-induced agranulocytosis (Manu et al., 2016). However, it has been argued that these guidelines ignore evidence that individuals with BEN may be safely treated with clozapine (Manu et al., 2016). Interestingly, no study adjusted for BEN in their analysis.

In the UK, some studies reported lower odds of clozapine prescription for Asian service-users compared to White service-users (Cho et al., 2019; Govind et al., 2020), whilst other studies reported no difference (Das-Munshi et al., 2018; Kesserwani et al., 2019). These mixed findings may be due to heterogeneity in samples, data sources and the aggregation of data from different Asian communities. Furthermore, previous research has shown that clozapine prescription practices differ across geographical locations in the UK (Downs & Zinkler, 2007).

In New Zealand, the higher percentage of Māori service-users receiving clozapine compared to European service-users (Dey et al., 2016; Wheeler, 2008; Wheeler et al., 2008), has been attributed to efforts specifically aimed at ensuring equality in access to mental health services and treatments for Māori and Pacific Islander communities (The Mental Health Commission, 1998; Wheeler et al., 2008), suggesting that such interventions can reduce ethnic disparities with clozapine.

Several studies (Copeland et al., 2003; Das-Munshi et al., 2018; Kelly et al., 2006; Mallinger et al., 2006; Manuel et al., 2012; Stroup et al., 2014) have attributed the underusage of clozapine amongst ethnic minority service-users to clinician fear over clozapine-increased risk of diabetes and CVD, where the risk of developing such health disorders are increased for those of minoritised ethnic backgrounds (Black, 2002; Spanakis & Golden, 2013). Only one study adjusted for these health conditions in analysis and ethnic disparities were found to persist (Stroup et al., 2014).

Multiple studies (Kelly et al., 2006; Kreyenbuhl et al., 2003; Kuno & Rothbard, 2002; Velligan et al., 2014) reported possible clinician assumption that minority ethnic service-users are less adherent to the frequent blood monitoring procedures which clozapine treatment demands may contribute to the underutilisation of clozapine in minority ethnic communities. These studies did not present this explanation with supporting evidence, and no study controlled for these factors in analysis. Recent systematic reviews have reported both clinician inexperience prescribing clozapine and patients' refusal of care (i.e. declining clozapine treatment due to its adverse side-effects or the need for intense blood monitoring), could be possible reasons for the disparities observed (Farooq et al., 2019; Verdoux et al., 2018). However, the studies reviewed did not address whether these reasons were driving the observed disparities. Further research is needed to clarify any impact of these factors.

It is important to observe that half of the included studies were not originally designed to analyse ethnic disparities in clozapine treatment, and any ethnic differences in the prevalence of TRS may confound the association between ethnicity and clozapine prescription in studies where the cohort is not restricted to TRS samples (i.e. confounding by indication). In this regard, one study reported that White European ethnicity (compared to Non-White European ethnicity) has been identified as a risk factor for TRS (Teo, Borlido, Kennedy, & De Luca, 2013), but other studies analysing predictors of TRS do not support this (Ajnakina et al., 2016; Bani-Fatemi et al., 2019; Demjaha et al., 2017). A minority of the included studies controlled for TRS in analysis (Beck et al., 2019; Cho et al., 2019; Das-Munshi et al., 2018; Stokes et al., 2020; Stroup et al., 2014), and ethnic disparities were found to persist in most of these studies. We observed that the patterns of ethnic disparities do not seem to be modified by TRS, which suggests that it is not the over or under-identification of TRS that prompts ethnic differences in access to clozapine.

Finally, it is important to discuss that racist and oppressive systems may contribute to the ethnic disparities observed. Regarding general health, implicit biases against Black, Latino and individuals with darker complexions/skin tones were found to be significantly related to treatment decisions, patient-provider interactions and health outcomes (Hall et al., 2015). The processes related to racism have resulted in differential access to resources and socio-economic opportunities, consequently leading to the experience of inequalities across a range of health outcomes, including access to mental health services and treatments (Nazroo, Bhui, & Rhodes, 2020; Williams & Mohammed, 2013). Systematic reviews have reported that minority ethnic groups with psychosis, particularly Black people, are more likely to experience differential pathways to mental health treatment, which are often complex and coercive compared to White service-users (Barnett et al., 2019; Bhui et al., 2003). Furthermore, it is important to acknowledge the challenges in capturing how racism adversely affects health, with the difficulty in defining specific measures for racism (Williams & Mohammed, 2013).

### Strengths and limitations of this review

This review addressed several gaps in the current literature. This review is the first to investigate whether ethnic disparities in clozapine prescription are observed internationally. This present study is the largest (having included a greater number of studies compared to previous works (Williams et al., 2020)), including studies conducted in Brazil, New Zealand and the UK, and has been able to synthesise data from the past three decades, including the most recent years (1991–2020).

The conclusions drawn in this review should be considered given the following limitations. We reported findings using the same ethnic terminology used by the authors of the papers included in the review. There has been criticism over the use of broad ethnic categories, which may integrate heterogeneous groups of people (Bhopal, 2004). The lack of specificity and amalgamation of ethnic groups together under one category runs the risk of merging very distinct experiences of people from various cultural settings and contributing to the underrepresentation of certain groups in the literature. Importantly, the use of broad classifications may cover up potential disparities affecting some ethnic communities and impair our understanding of ethnic differences in treatments and disease prevalence (Bhopal, 2004). Some of the included studies merge different groups under one category (Beck et al., 2019; de Almeida et al., 2020; Dey et al., 2016; Mark, Palmer, Russo, & Vasey, 2003). Caution should be taken when interpreting these findings. Moreover, the search strategy was written in English and constructed using ethnicity terminology from five Western, industrialised, English-speaking countries (i.e. Australia, Canada, New Zealand, the UK, and the USA), so this review may be more likely to identify studies from these countries (and any other countries also using the same ethnic terminology) and not from others. There is limited generalisability of the results and implications beyond the four countries investigated in this present study. Except for the results from Brazil, most of the findings are weighted towards English-speaking, Western countries. Therefore, the conclusions drawn from this review are subject to a Eurocentric focus and may not be reflective of clozapine prescription practices, guidelines, and the experiences of minority ethnic groups in other countries. Caution should be taken when attempting to generalise to other cultural settings.

### Implications and future research

The results of this review suggest that access to clozapine treatment varies among ethnic groups in certain countries. This raises an ethical concern, given that clozapine is the recommended medication for TRS patients (Farooq et al., 2019). Clozapine treatment has been associated with reduced risk of hospital readmission (Kesserwani et al., 2019) and lower mortality rates (Vermeulen et al., 2019), leaving some ethnic groups with a higher risk of experiencing such negative outcomes. Disparities in access to clozapine could be regarded as a health inequity, where people of specific ethnic backgrounds have less opportunity to achieve optimal health status (World Health Organisation, 1946, 2017, 2018). This elucidates why ethnic disparities in access to clozapine is a major public health issue that needs to be addressed. The failure to overcome health inequities infringe upon human rights regarding fairness and equity in health (World Health Organisation, 2018).

Healthcare systems and policies may benefit from interventions targeted towards ethnic minority communities. In New Zealand, there have been efforts to actively increase the involvement of the Māori and Pacific Islander people in the planning and delivery of mental health services with the aim of promoting culturally sensitive care (Ministry of Health, 2002). Studies have shown that patient-clinician matching of language and ethnicity improve patient-clinician relationships, encourages antipsychotic medication compliance (Ziguras, Klimidis, Lambert, & Jackson, 2001) and is associated with length and outcome of treatment (Sue, Fujino, Hu, Takeuchi, & Zane, 1991).

Some of the frequent reasons possibly explaining the observed disparities were not analysed in many studies. Future studies should investigate the impact of BEN, comorbid health conditions and potential compliance with blood monitoring with clozapine initiation to affirm whether ethnic differences in clozapine prescription could be accounted for by these dynamics.

## Conclusion

This review synthesised the international literature regarding ethnic disparities in clozapine prescription in cohorts of individuals with schizophrenia-spectrum disorders. The results suggest that Black and Hispanic service-users in the UK and the USA are significantly less likely to receive clozapine than White/Caucasian service-users. A better understanding of possible driving factors behind ethnic disparities may help better inform interventions and policy reforms to promote equal access to schizophrenia treatments. Evidence from studies in New Zealand suggests that interventions that promote culturally sensitive care can reduce ethnic disparities with clozapine.

## Supplementary material

For supplementary material accompanying this paper visit https://doi.org/10.1017/S0033291722001878. click here to view supplementary material
