# Supplementary material for: Ethnic disparities in clozapine prescription for service-users with schizophrenia-spectrum disorders: a systematic review
Source: Psychol Med. 2022 Jul 5;52(12):2212–23. doi: 10.1017/S0033291722001878 (PMC9527670; doi:10.1017/S0033291722001878)
Supplement: Supplementary file 1 [file S0033291722001878sup001.docx]

**Ethnic Disparities in Clozapine Prescription for Service-users with Schizophrenia-Spectrum Disorders: A Systematic Review**

Supplementary Material

**Anita Margarette Bayya Ventura** ^a^**,**

**Dr Richard D. Hayes** ^a^**, Dr Daniela Fonseca de Freitas** ^a,b^ *****

^a^ Department of Psychological Medicine, Institute of Psychiatry, Psychology & Neuroscience, King’s College London, London, United Kingdom

^b^ Department of Psychiatry, University of Oxford, Oxford, United Kingdom

*Correspondence should be addressed to: Daniela Fonseca de Freitas, Institute of Psychiatry, Psychology & Neuroscience, King’s College London, 16 De Crespigny Park, Denmark Hill, London, SE5 8AF, United Kingdom. E-mail: [daniela.fonseca_de_freitas@kcl.ac.uk](mailto:daniela.fonseca_de_freitas@kcl.ac.uk)

**Supplement Table 1:** *Summary of Records Retrieved from each Database*

| **Database** | **Number of Records Retrieved** |
| --- | --- |
| CINAHL | 190 |
| PubMed | 504 |
| Medline | 53 |
| Embase | 481 |
| PsycINFO | 319 |
| Open Grey | 0 |
| **Total** | **1547** |
| **Deduplicated^a^** | **1040** |

*Notes.* All databases searched on December 13^th^, 2020.

^a^Number of studies from six electronic databases only, with duplicates removed, to undergo two-stage screening process

**Supplement Table 2:** *Search Terms and Number of Records for CINAHL*

| **Number** | **CINAHL via EBSCO Host Service** |  |
| --- | --- | --- |
|  | **Search Term** | **Results** |
| **1** | AB ("clozapine prescription" or "clozapine prescribing" or "clozapine prescri*" or "prescription of clozapine" or "prescriptions of clozapine" or "prescri* of clozapine" or "prescribing of clozapine" or "use of clozapine" or "uses of clozapine" or "usage of clozapine" or "usages of clozapine" or "clozapine use" or "clozapine-use" or "clozapine uses" or "clozapine usage" or "clozapine usages" or "utilisation of clozapine" or "utilization of clozapine" or "utilisations of clozapine" or "utilizations of clozapine" or "utili* of clozapine" or "clozapine utili*" or "clozapine utilisation" or "clozapine utilization" or "clozapine utilisations" or "clozapine utilizations" or "utilising clozapine" or "utilizing clozapine" or "utility of clozapine" or "clozapine initiation" or "clozapine initia*" or "initiation of clozapine" or "initia* of clozapine" or "initiating clozapine") | 207 |
| **2** | AB (clozapine or zaponex or denzapin* or clozaril or leponex) | 1,557 |
| **3** | AB (prescription or prescribing or prescri* or "prescription of" or "prescriptions of" or "prescri* of" or "prescribing of" or "use of" or "uses of" or "usage of" or "usages of" or uses or usage O usages or "utilisation of" or "utilization of" or "utilisations of" or "utilizations of" or "utili* of" or utilisation or utilization or utilisations or utilizations or utilising or utilizing or utility or "utility of" or initiation or intia* or "initiations of" or initiating) | 861,026 |
| **4** | TX (refugee* OR immigrant* OR (asyl* adj1 seek*) OR foreign* OR ethnic* OR "ethnic groups" OR "ethnic-groups" OR "minority groups" OR "population groups" OR minorit* OR race OR racial* OR multiethnic* OR multi-ethnic* OR nationalit* OR nation* OR "foreign-national" or "foreign national" OR "non-white" OR "non white" OR "non-national" OR "non national" OR tribe*) | 929,265 |
| **5** | (MH "Refugees") OR (MH "Immigrants") OR (MH "Transients and Migrants") | 25,751 |
| **6** | (MH "Ethnic Groups") OR (MH "Minority Groups") | 37,596 |
| **7** | TX (indig* OR aborig* OR "first people*" OR "first australian*" OR "koori*" OR "goori*" OR "first-australian*" OR native* OR "native american*" OR "native-american*" OR inuit* OR maori OR niuean*) | 53,375 |
| **8** | (MH "Native Americans") OR (MH "Indigenous Peoples") OR (MH "Aboriginal Canadians") OR (MH "First Nations of Canada") OR (MH "Inuit") OR (MH "First Nations of Australia") OR (MH "Aboriginal Australians") OR (MH "Torres Strait Islanders") | 18,638 |
| **9** | TX (black OR "black british" OR "black-british" OR "african american" OR "african-american" OR african* OR caribbean* OR afro-caribbean* OR afrocaribbean* OR "afro-latin*" OR "afrolatin* OR afro* OR "BAME" OR "BME") | 132,440 |
| **10** | (MH "Blacks") | 2 |
| **11** | TX (asian OR "asian british" OR "asian-british" OR "asian american*" OR "asian-american*" OR indian* OR pakistani* OR bangladeshi* OR chinese or korean OR japanese OR filipin* OR viet* "asian-indian" OR "asian indian") | 318,485 |
| **12** | (MH "Asians") OR (MH "Cambodians") OR (MH "Chinese") OR (MH "Filipinos") OR (MH "Hmong") OR (MH "Japanese") OR (MH "Koreans") OR (MH "Laotians") OR (MH "Thai") OR (MH "Vietnamese") | 32,759 |
| **13** | TX (native* OR "native hawaiian" OR "native-hawaiian" OR samoan* OR chamorro* OR tonga* OR fiji* OR marshallese* OR tahiti* OR "pacific islander" OR "pacific-islander") | 27,787 |
| **14** | (MH "Maori") OR (MH "Indigenous Peoples") | 4,726 |
| **15** | TX (latin* or hispan* or arab* or arabic* or "middle-east*" or "Middle East*") | 185,810 |
| **16** | (MH "Amish") OR (MH "Arabs") OR (MH "Hispanics") OR (MH "Jews") OR (MH "Kurds") | 4,124 |
| **17** | or/4-16 | 1,383,208 |
| **18** | 2 AND 3 | 743 |
| **19** | 1 OR 18 | 744 |
| **20** | 19 AND 17 | 190 |

**Supplement Table 3:** *Search Terms and Number of Records for PubMed*

| **Number** | **PubMed via National Centre of Biotechnology Information (NCBI)** |  |
| --- | --- | --- |
|  | **Search Terms** | **Results** |
| **1** | Clozapine[Title/Abstract] OR zaponex[Title/Abstract] OR denzapin*[Title/Abstract] OR clozaril[Title/Abstract] OR leponex[Title/Abstract] | 11,936 |
| **2** | Prescription[Title/Abstract] OR prescribing[Title/Abstract] OR prescriptions[Title/Abstract] OR "prescription of"[Title/Abstract] OR "prescriptions of"[Title/Abstract] OR use[Title/Abstract] OR usage[Title/Abstract] OR "use of"[Title/Abstract] OR "usage of"[Title/Abstract] OR utilisation[Title/Abstract] OR utilization[Title/Abstract] OR "utilisation of"[Title/Abstract] OR "utilization of"[Title/Abstract] OR "utilisations"[Title/Abstract] OR "utilizations"[Title/Abstract] OR "utilising"[Title/Abstract] OR "utilizing"[Title/Abstract] OR "utility of"[Title/Abstract] OR initiation[Title/Abstract] OR initiations[Title/Abstract] OR initiating[Title/Abstract] OR "initiation of"[Title/Abstract] | 3,666,049 |
| **3** | 1 AND 2 | 2,956 |
| **4** | ("Clozapine prescription"[Title/Abstract] OR "clozapine prescribing"[Title/Abstract] OR "clozapine prescri*"[Title/Abstract] OR "clozapine use"[Title/Abstract] OR "clozapine-use"[Title/Abstract] OR "clozapine usage"[Title/Abstract] OR "clozapine utili*"[Title/Abstract] OR "clozapine initiation"[Title/Abstract] OR "clozapine initia*"[Title/Abstract]) | 376 |
| **5** | ("clozapine prescription"[Title/Abstract] OR "clozapine prescribing"[Title/Abstract] OR "clozapine prescri*"[Title/Abstract] OR prescription n3 clozapine[Title/Abstract] OR prescriptions n3 clozapine[Title/Abstract] OR prescri* n3 clozapine[Title/Abstract] OR prescribing n3 clozapine[Title/Abstract] OR uses n3 clozapine[Title/Abstract] OR usage n3 clozapine[Title/Abstract] OR usages n3 clozapine[Title/Abstract] OR "clozapine use"[Title/Abstract] OR "clozapine-use"[Title/Abstract] OR clozapine n3 uses[Title/Abstract] OR "clozapine usage"[Title/Abstract] OR clozapine n3 usages[Title/Abstract] OR utilisation n3 clozapine[Title/Abstract] OR utilization n3 clozapine[Title/Abstract] OR utilisations n3 clozapine[Title/Abstract] OR utilizations n3 clozapine[Title/Abstract] OR utili* n3 clozapine[Title/Abstract] OR "clozapine utili*"[Title/Abstract] OR clozapine n3 utilisation[Title/Abstract] OR clozapine n3 utilization[Title/Abstract] OR clozapine n3 utilisations[Title/Abstract] OR clozapine n3 utilizations[Title/Abstract] OR utilising n3 clozapine[Title/Abstract] OR utilizing n3 clozapine[Title/Abstract] OR utility n3 clozapine[Title/Abstract] OR "clozapine initiation"[Title/Abstract] OR "clozapine initia*"[Title/Abstract] OR initiation n3 clozapine[Title/Abstract] OR initia* n3 clozapine[Title/Abstract] OR "initiating clozapine"[Title/Abstract]) | 408 |
| **6** | 3 OR 4 OR 5 | 2,965 |
| **7** | (refugee* OR immigrant* OR (asyl* adj1 seek*) OR foreign* OR ethnic* OR "ethnic groups" OR "ethnic-groups" OR "minority groups" OR "population groups" OR minorit* OR race OR racial* OR multiethnic* OR multi-ethnic* OR nationalit* OR nation* OR "foreign-national" or "foreign national" OR "non-white" OR "non white" OR "non-national" OR "non national" OR tribe*) | 2,702,718 |
| **8** | (indig* OR aborig* OR "first people*" OR "first australian*" OR "koori*" OR "goori*" OR "first-australian*" OR native* OR "native american*" OR "native-american*" OR inuit* OR maori OR niuean*) | 302,911 |
| **9** | (black OR "black british" OR "black-british" OR "african american" OR "african-american" OR african* OR caribbean* OR afro-caribbean* OR afrocaribbean* OR afro* OR "BAME" OR "BME") | 378,632 |
| **10** | (asian OR "asian british" OR "asian-british" OR "asian american*" OR "asian-american*" OR indian* OR pakistani* OR bangladeshi* OR chinese or korean OR japanese OR filipin* OR viet* "asian-indian" OR "asian indian") | 1,514 |
| **11** | (native* OR "native hawaiian" OR "native-hawaiian" OR samoan* OR chamorro* OR tonga* OR fiji* OR marshallese* OR tahiti* OR "pacific islander" OR "pacific-islander") | 231,401 |
| **12** | (latin* or hispan* or arab* or arabic* or "middle-east*" or "Middle East*") | 370,961 |
| **13** | or/7-12 | 3,407,793 |
| **14** | **(((((((((((((((((((((((("Refugees"[Mesh]) OR "Emigrants and Immigrants"[Mesh:NoExp]) OR "Ethnic Groups"[Mesh])) OR "African Continental Ancestry Group"[Mesh]) OR "African Americans"[Mesh]) OR "American Native Continental Ancestry Group"[Mesh]) OR "Alaska Natives"[Mesh]) OR "Indians, Central American"[Mesh]) OR "Indians, North American"[Mesh]) OR "Indians, South American"[Mesh]) OR "Inuits"[Mesh]) OR "Asian Continental Ancestry Group"[Mesh]) OR "Asian Americans"[Mesh]) OR "Oceanic Ancestry Group"[Mesh]) OR "Minority Groups"[Mesh]) OR "Amish"[Mesh]) OR "Arabs"[Mesh]) OR "Hispanic Americans"[Mesh]) OR "Mexican Americans"[Mesh]) OR "Indigenous Peoples"[Mesh]) OR "Jews"[Mesh]) OR "Roma"[Mesh]) OR "Continental Population Groups"[Mesh:NoExp])** | 308,340 |
| **15** | 13 OR 14 | 3,413,929 |
| **16** | 6 AND 15 | 504 |

**Supplement Table 4:** *Search Terms and Number of Records for Medline*

| **Number** | **Medline via Ovid** |  |
| --- | --- | --- |
|  | **Search Terms** | **Results** |
| **1** | (Clozapine or zaponex or denzapin* or clozaril or leponex).ab. | 9,989 |
| **2** | (prescription or prescribing or prescri* or "prescription of" or "prescriptions of" or "prescri* of" or "prescribing of" or "use of" or "uses of" or "usage of" or "usages of" or uses or usage O usages or "utilisation of" or "utilization of" or "utilisations of" or "utilizations of" or "utili* of" or utilisation or utilization or utilisations or utilizations or utilising or utilizing or utility or "utility of" or initiation or intia* or "initiations of" or initiating).ab. | 3,701,798 |
| **3** | ("clozapine prescription" or "clozapine prescribing" or "clozapine prescri*" or "prescription of clozapine" or "prescriptions of clozapine" or "prescri* of clozapine" or "prescribing of clozapine" or "use of clozapine" or "uses of clozapine" or "usage of clozapine" or "usages of clozapine" or "clozapine use" or "clozapine-use" or "clozapine uses" or "clozapine usage" or "clozapine usages" or "utilisation of clozapine" or "utilization of clozapine" or "utilisations of clozapine" or "utilizations of clozapine" or "utili* of clozapine" or "clozapine utili*" or "clozapine utilisation" or "clozapine utilization" or "clozapine utilisations" or "clozapine utilizations" or "utilising clozapine" or "utilizing clozapine" or "utility of clozapine" or "clozapine initiation" or "clozapine initia*" or "initiation of clozapine" or "initia* of clozapine" or "initiating clozapine").ab. | 861 |
| **4** | Minority Groups.mp. | 17,537 |
| **5** | Minority Groups/ or (group, minority or groups, minority or minority group or minority groups).mp. | 18,516 |
| **6** | Ethnic Groups.mp. | 83,484 |
| **7** | Ethnic Groups/ or (ethnic group or ethnic groups or ethnicity or nationality).mp. | 145,877 |
| **8** | (refugee* or immigrant* or (asyl* adj1 seek*) or foreign* or ethnic* or minorit* or race or racial* or multiethnic* or multi-ethnic* or nationalit* or nation* or "foreign-national" or "foreign national" or "non-white" or "non white" or "non-national" or "non national" or tribe*).mp. | 1,111,658 |
| **9** | Refugees/ or (asylum seeker or asylum seeker, political or asylum seekers or asylum seekers, political or displaced person or displaced person, internally or displaced persons or displaced persons, internally or internally displaced person or internally displaced persons or person, displaced or persons, displaced or political asylum seeker or political asylum seekers or political refugee or political refugees or refugee or refugee, political or refugees or refugees, political or seeker, asylum or seekers, asylum or seekers, political asylum).mp. | 15,281 |
| **10** | Emigrants and Immigrants"/ or ((((alien or aliens or emigrant or emigrants or emigrants) and immigrants) or foreigner or foreigners or immigrant or immigrants or immigrants) and emigrants).mp. | 12,719 |
| **11** | Continental Population Groups/ or (continental population group or continental population groups or group, continental population or groups, continental population or population group, continental or population groups, continental or race or races).mp. | 133,276 |
| **12** | (indig* or aborig* or "first people*" or "first australian*" or "first-australian*" or "koori*" or "goori*" or native* or "native american*" or "native-american*" or inuit* or maori or niuean*).mp. | 281,415 |
| **13** | American Native Continental Ancestry Group/ or (Alaska Natives/ or Indians, Central American/ or Indians, North American/ or Indians, South American/ or Inuits/) or american native continental ancestry group.mp. or (alaska indigenous people or alaska indigenous peoples or alaska native or alaska natives or alaska's indigenous people or alaska's indigenous peoples or alaskas indigenous people or alaskian, native or alaskians, native or indigenous people, alaska's or indigenous people of alaska or indigenous peoples, alaska's or native, alaska or native alaskian or native alaskians or natives, alaska or people, alaska's indigenous or peoples, alaska's indigenous).mp. or (american amerind, central or american amerinds, central or american indian, central or american indians, central or amerind, central american or amerinds, central american or central american amerind or central american amerinds or central american indian or central american indians or indian, central american or indians, central american).mp. or (american indian or american indian, north or american indians or american indians, north or american, native or americans, native or amerind, north american or amerinds, north american or indian, american or indian, north american or indians, american or indians, north american or native american or native americans or north american amerind or north american amerinds or north american indian or north american indians).mp. or (american amerind, south or american amerinds, south or american indian, south or american indians, south or amerind, south american or amerinds, south american or indian, south american or indians, south american or south american amerind or south american amerinds or south american indian or south american indians).mp. or (aleut or aleuts or inuit or inuits or inupiat or inupiats or kalaallit or kalaallits).mp. | 28,243 |
| **14** | Indigenous Peoples/ or (first nation people or first nation peoples or indigenous people or indigenous peoples or nation people, first or nation peoples, first or native people or native peoples or people, first nation or people, indigenous or people, native or peoples, first nation or peoples, indigenous or peoples, native).mp. or Population Groups/ or (group, population or groups, population or indigenous population or indigenous populations or native born or native-born or natives or population group or population groups or population, indigenous or populations, indigenous or tribes).mp. | 55,446 |
| **15** | (black or "black british" or "black-british" or "african american" or "african-american" or african* or caribbean* or afro-caribbean* or afrocaribbean* or afro* or "BAME" or "BME").mp. | 322,111 |
| **16** | African Continental Ancestry Group/ or (african continental ancestry group or blacks).mp. or African Americans/ or (african americans or african-american or african-americans).mp. | 123,372 |
| **17** | (asian or "asian british" or "asian-british" or "asian american*" or "asian-american*" or indian* or pakistani* or bangladeshi* or chinese or korean or japanese or filipin* or "viet* asian-indian" or "asian indian").mp. | 631,556 |
| **18** | Asian Continental Ancestry Group/ or (asian or asian continental ancestry group or asians or asiatic race or asiatic races or burmese or burmeses or cambodian or cambodians or chinese or japanese or koreans or thai or thaus or vietnamese or vietnameses).mp. or Asian Americans/ or (american, cambodian or american, korean or american, vietnamese or americans, asian or americans, cambodian or americans, chinese or americans, filipino or americans, hmong or americans, japanese or americans, korean or americans, vietnamese or asian american or asian americans or asian indian american or asian indian americans or cambodian american or cambodian americans or chinese american or chinese americans or filipino american or filipino americans or hmong american or hmong americans or indian american, asian or japanese american or japanese americans or korean american or korean americans or vietnamese american or vietnamese americans).mp. | 521,493 |
| **19** | (native* or "native hawaiian" or "native-hawaiian" or samoan* or chamorro* or tonga* or fiji* or marshallese* or tahiti* or "pacific islander" or "pacific-islander").mp. | 228,493 |
| **20** | Oceanic Ancestry Group/ or (aborigine, australian or aborigines, australian or ancestry group, oceanic or ancestry groups, oceanic or australian aborigine or australian aborigines or australian race or oceanic ancestry or groups, oceanic ancestry or hawaiian, native or hawaiians, native or native hawaiian or native hawaiians or oceanic ancestry group or oceanic ancestry groups or pacific island american or pacific island americans or pacific islander american or pacific islander americans or race, australian or races, australian or races).mp. | 11,627 |
| **21** | (latin* or hispan* or arab* or arabic* or "middle-east*" or "Middle East*").mp. | 272,326 |
| **22** | Hispanic Americans/ or Mexican Americans/ or (american, hispanic or americans, cuban or americans, hispanic or americans, spanish or cuban american or cuban americans or hispanic or hispanic american or hispanic americans or hispanics or latina or latinas or latino or latinos or puerto rican or puerto ricans or spanish american or spanish americans).mp. or (american, mexican or americans, mexican or chicana or chicanas or mexican american or mexican americans).mp. | 72,200 |
| **23** | Amish/ or Arabs/ or Roma/ or Jews/ or (arab or arabs or bedouin or bedouins or palestinian or palestinians).mp. or (roma or romani).mp. or (jew or jews).mp. | 29,749 |
| **24** | or/4-23 | 2,343,176 |
| **25** | 1 AND 2 | 3,075 |
| **26** | 3 OR 25 | 3,089 |
| **27** | 24 AND 26 | 53 |

**Supplement Table 5:** *Search Terms and Number of Records for Embase*

| **Number** | **Embase via Ovid** |  |
| --- | --- | --- |
|  | **Search Terms** | **Results** |
| **1** | (Clozapine or zaponex or denzapin* or clozaril or leponex).ab. | 14,155 |
| **2** | (prescription or prescribing or prescri* or "prescription of" or "prescriptions of" or "prescri* of" or "prescribing of" or "use of" or "uses of" or "usage of" or "usages of" or uses or usage O usages or "utilisation of" or "utilization of" or "utilisations of" or "utilizations of" or "utili* of" or utilisation or utilization or utilisations or utilizations or utilising or utilizing or utility or "utility of" or initiation or intia* or "initiations of" or initiating).ab. | 4,933,193 |
| **3** | ("clozapine prescription" or "clozapine prescribing" or "clozapine prescri*" or "prescription of clozapine" or "prescriptions of clozapine" or "prescri* of clozapine" or "prescribing of clozapine" or "use of clozapine" or "uses of clozapine" or "usage of clozapine" or "usages of clozapine" or "clozapine use" or "clozapine-use" or "clozapine uses" or "clozapine usage" or "clozapine usages" or "utilisation of clozapine" or "utilization of clozapine" or "utilisations of clozapine" or "utilizations of clozapine" or "utili* of clozapine" or "clozapine utili*" or "clozapine utilisation" or "clozapine utilization" or "clozapine utilisations" or "clozapine utilizations" or "utilising clozapine" or "utilizing clozapine" or "utility of clozapine" or "clozapine initiation" or "clozapine initia*" or "initiation of clozapine" or "initia* of clozapine" or "initiating clozapine").ab. | 1,313 |
| **4** | Minority Groups.mp. | 5,658 |
| **5** | Minority Groups/ or (group, minority or groups, minority or minority group or minority groups).mp. | 19,433 |
| **6** | Ethnic Groups.mp. | 35,591 |
| **7** | Ethnic Groups/ or (ethnic group or ethnic groups or ethnicity or nationality).mp. | 213,060 |
| **8** | (refugee* or immigrant* or (asyl* adj1 seek*) or foreign* or ethnic* or minorit* or race or racial* or multiethnic* or multi-ethnic* or nationalit* or nation* or "foreign-national" or "foreign national" or "non-white" or "non white" or "non-national" or "non national" or tribe*).mp. | 1,587,955 |
| **9** | Refugees/ or (asylum seeker or asylum seeker, political or asylum seekers or asylum seekers, political or displaced person or displaced person, internally or displaced persons or displaced persons, internally or internally displaced person or internally displaced persons or person, displaced or persons, displaced or political asylum seeker or political asylum seekers or political refugee or political refugees or refugee or refugee, political or refugees or refugees, political or seeker, asylum or seekers, asylum or seekers, political asylum).mp. | 17,262 |
| **10** | Emigrants and Immigrants"/ or ((((alien or aliens or emigrant or emigrants or emigrants) and immigrants) or foreigner or foreigners or immigrant or immigrants or immigrants) and emigrants).mp. | 6,524 |
| **11** | Continental Population Groups/ or (continental population group or continental population groups or group, continental population or groups, continental population or population group, continental or population groups, continental or race or races).mp. | 220,001 |
| **12** | (indig* or aborig* or "first people*" or "first australian*" or "first-australian*" or "koori*" or "goori*" or native* or "native american*" or "native-american*" or inuit* or maori or niuean*).mp. | 329,314 |
| **13** | American Native Continental Ancestry Group/ or (Alaska Natives/ or Indians, Central American/ or Indians, North American/ or Indians, South American/ or Inuits/) or american native continental ancestry group.mp. or (alaska indigenous people or alaska indigenous peoples or alaska native or alaska natives or alaska's indigenous people or alaska's indigenous peoples or alaskas indigenous people or alaskian, native or alaskians, native or indigenous people, alaska's or indigenous people of alaska or indigenous peoples, alaska's or native, alaska or native alaskian or native alaskians or natives, alaska or people, alaska's indigenous or peoples, alaska's indigenous).mp. or (american amerind, central or american amerinds, central or american indian, central or american indians, central or amerind, central american or amerinds, central american or central american amerind or central american amerinds or central american indian or central american indians or indian, central american or indians, central american).mp. or (american indian or american indian, north or american indians or american indians, north or american, native or americans, native or amerind, north american or amerinds, north american or indian, american or indian, north american or indians, american or indians, north american or native american or native americans or north american amerind or north american amerinds or north american indian or north american indians).mp. or (american amerind, south or american amerinds, south or american indian, south or american indians, south or amerind, south american or amerinds, south american or indian, south american or indians, south american or south american amerind or south american amerinds or south american indian or south american indians).mp. or (aleut or aleuts or inuit or inuits or inupiat or inupiats or kalaallit or kalaallits).mp. | 27,733 |
| **14** | Indigenous Peoples/ or (first nation people or first nation peoples or indigenous people or indigenous peoples or nation people, first or nation peoples, first or native people or native peoples or people, first nation or people, indigenous or people, native or peoples, first nation or peoples, indigenous or peoples, native).mp. or Population Groups/ or (group, population or groups, population or indigenous population or indigenous populations or native born or native-born or natives or population group or population groups or population, indigenous or populations, indigenous or tribes).mp. | 37,768 |
| **15** | (black or "black british" or "black-british" or "african american" or "african-american" or african* or caribbean* or afro-caribbean* or afrocaribbean* or afro* or "BAME" or "BME").mp. | 400,971 |
| **16** | African Continental Ancestry Group/ or (african continental ancestry group or blacks).mp. or African Americans/ or (african americans or african-american or african-americans).mp. | 143,548 |
| **17** | (asian or "asian british" or "asian-british" or "asian american*" or "asian-american*" or indian* or pakistani* or bangladeshi* or chinese or korean or japanese or filipin* or viet* asian-indian" or "asian indian").mp. | 893,121 |
| **18** | Asian Continental Ancestry Group/ or (asian or asian continental ancestry group or asians or asiatic race or asiatic races or burmese or burmeses or cambodian or cambodians or chinese or japanese or koreans or asiatic or race, thai or thaus or vietnamese or vietnameses).mp. or Asian Americans/ or (american, cambodian or american, korean or american, vietnamese or americans, asian or americans, cambodian or americans, chinese or americans, filipino or americans, hmong or americans, japanese or americans, korean or americans, vietnamese or asian american or asian americans or asian indian american or asian indian americans or cambodian american or cambodian americans or chinese american or chinese americans or filipino american or filipino americans or hmong american or hmong americans or indian american, asian or japanese american or japanese americans or korean american or korean americans or vietnamese american or vietnamese americans).mp. | 733,303 |
| **19** | (native* or "native hawaiian" or "native-hawaiian" or samoan* or chamorro* or tonga* or fiji* or marshallese* or tahiti* or "pacific islander" or "pacific-islander").mp. | 267,150 |
| **20** | Oceanic Ancestry Group/ or (aborigine, australian or aborigines, australian or ancestry group, oceanic or ancestry groups, oceanic or australian aborigine or australian aborigines or australian race or oceanic ancestry or groups, oceanic ancestry or hawaiian, native or hawaiians, native or native hawaiian or native hawaiians or oceanic ancestry group or oceanic ancestry groups or pacific island american or pacific island americans or pacific islander american or pacific islander americans or race, australian or races, australian or races).mp. | 4,880 |
| **21** | (latin* or hispan* or arab* or arabic* or "middle-east*" or "Middle East*").mp. | 330,987 |
| **22** | Hispanic Americans/ or Mexican Americans/ or (american, hispanic or americans, cuban or americans, hispanic or americans, spanish or cuban american or cuban americans or hispanic or hispanic american or hispanic americans or hispanics or latina or latinas or latino or latinos or puerto rican or puerto ricans or spanish american or spanish americans).mp. or (american, mexican or americans, mexican or mexican american or mexican americans).mp. | 106,685 |
| **23** | Amish/ or Arabs/ or Roma/ or Jews/ or (arab or arabs or bedouin or bedouins or palestinian or palestinians).mp. or (roma or romani).mp. or (jew or jews).mp. | 38,551 |
| **24** | or/4-23 | 3,121,844 |
| **25** | 1 AND 2 | 4,798 |
| **26** | 3 OR 25 | 4,818 |
| **27** | 24 AND 26 | 481 |

**Supplement Table 6:** *Search Terms and Number of Records for APA PsycINFO*

| **Number** | **APA PsycINFO via Ovid** |  |
| --- | --- | --- |
|  | **Search Terms** | **Results** |
| **1** | (Clozapine or zaponex or denzapin* or clozaril or leponex).ab. | 7,885 |
| **2** | (prescription or prescribing or prescri* or "prescription of" or "prescriptions of" or "prescri* of" or "prescribing of" or "use of" or "uses of" or "usage of" or "usages of" or uses or usage O usages or "utilisation of" or "utilization of" or "utilisations of" or "utilizations of" or "utili* of" or utilisation or utilization or utilisations or utilizations or utilising or utilizing or utility or "utility of" or initiation or intia* or "initiations of" or initiating).ab. | 965,758 |
| **3** | ("clozapine prescription" or "clozapine prescribing" or "clozapine prescri*" or "prescription of clozapine" or "prescriptions of clozapine" or "prescri* of clozapine" or "prescribing of clozapine" or "use of clozapine" or "uses of clozapine" or "usage of clozapine" or "usages of clozapine" or "clozapine use" or "clozapine-use" or "clozapine uses" or "clozapine usage" or "clozapine usages" or "utilisation of clozapine" or "utilization of clozapine" or "utilisations of clozapine" or "utilizations of clozapine" or "utili* of clozapine" or "clozapine utili*" or "clozapine utilisation" or "clozapine utilization" or "clozapine utilisations" or "clozapine utilizations" or "utilising clozapine" or "utilizing clozapine" or "utility of clozapine" or "clozapine initiation" or "clozapine initia*" or "initiation of clozapine" or "initia* of clozapine" or "initiating clozapine").ab. | 833 |
| **4** | Minority Groups.mp. | 21,472 |
| **5** | Minority Groups/ or (group, minority or groups, minority or minority group or minority groups).mp. | 23,124 |
| **6** | Ethnic Groups.mp. | 31,814 |
| **7** | Ethnic Groups/ or (ethnic group or ethnic groups or ethnicity or nationality).mp. | 71,390 |
| **8** | (refugee* or immigrant* or (asyl* adj1 seek*) or foreign* or ethnic* or minorit* or race or racial* or multiethnic* or multi-ethnic* or nationalit* or nation* or "foreign-national" or "foreign national" or "non-white" or "non white" or "non-national" or "non national" or tribe*).mp. | 469,584 |
| **9** | Refugees/ or (asylum seeker or asylum seeker, political or asylum seekers or asylum seekers, political or displaced person or displaced person, internally or displaced persons or displaced persons, internally or internally displaced person or internally displaced persons or person, displaced or persons, displaced or political asylum seeker or political asylum seekers or political refugee or political refugees or refugee or refugee, political or refugees or refugees, political or seeker, asylum or seekers, asylum or seekers, political asylum).mp. | 10,397 |
| **10** | Emigrants and Immigrants"/ or ((((alien or aliens or emigrant or emigrants or emigrants) and immigrants) or foreigner or foreigners or immigrant or immigrants or immigrants) and emigrants).mp. | 109 |
| **11** | Continental Population Groups/ or (continental population group or continental population groups or group, continental population or groups, continental population or population group, continental or population groups, continental or race or races).mp. | 76,221 |
| **12** | (indig* or aborig* or "first people*" or "first australian*" or "first-australian*" or "koori*" or "goori*" or native* or "native american*" or "native-american*" or inuit* or maori or niuean*).mp. | 47,992 |
| **13** | American Native Continental Ancestry Group/ or (Alaska Natives/ or Indians, Central American/ or Indians, North American/ or Indians, South American/ or Inuits/) or american native continental ancestry group.mp. or (alaska indigenous people or alaska indigenous peoples or alaska native or alaska natives or alaska's indigenous people or alaska's indigenous peoples or alaskas indigenous people or alaskian, native or alaskians, native or indigenous people, alaska's or indigenous people of alaska or indigenous peoples, alaska's or native, alaska or native alaskian or native alaskians or natives, alaska or people, alaska's indigenous or peoples, alaska's indigenous).mp. or (american amerind, central or american amerinds, central or american indian, central or american indians, central or amerind, central american or amerinds, central american or central american amerind or central american amerinds or central american indian or central american indians or indian, central american or indians, central american).mp. or (american indian or american indian, north or american indians or american indians, north or american, native or americans, native or amerind, north american or amerinds, north american or indian, american or indian, north american or indians, american or indians, north american or native american or native americans or north american amerind or north american amerinds or north american indian or north american indians).mp. or (american amerind, south or american amerinds, south or american indian, south or american indians, south or amerind, south american or amerinds, south american or indian, south american or indians, south american or south american amerind or south american amerinds or south american indian or south american indians).mp. or (aleut or aleuts or inuit or inuits or inupiat or inupiats or kalaallit or kalaallits).mp. | 13,100 |
| **14** | Indigenous Peoples/ or (first nation people or first nation peoples or indigenous people or indigenous peoples or nation people, first or nation peoples, first or native people or native peoples or people, first nation or people, indigenous or people, native or peoples, first nation or peoples, indigenous or peoples, native).mp. or Population Groups/ or (group, population or groups, population or indigenous population or indigenous populations or native born or native-born or natives or population group or population groups or population, indigenous or populations, indigenous or tribes).mp. | 16,186 |
| **15** | (black or "black british" or "black-british" or "african american" or "african-american" or african* or caribbean* or afro-caribbean* or afrocaribbean* or afro* or "BAME" or "BME").mp. | 126,849 |
| **16** | African Continental Ancestry Group/ or (african continental ancestry group or blacks).mp. or African Americans/ or (african americans or african-american or african-americans).mp. | 83,340 |
| **17** | (asian or "asian british" or "asian-british" or "asian american*" or "asian-american*" or indian* or pakistani* or bangladeshi* or chinese or korean or japanese or filipin* or "viet* asian-indian" or "asian indian").mp. | 144,449 |
| **18** | Asian Continental Ancestry Group/ or (asian or asian continental ancestry group or asians or asiatic race or asiatic races or burmese or burmeses or cambodian or cambodians or chinese or japanese or koreans or thai or thaus or vietnamese or vietnameses).mp. or Asian Americans/ or (american, cambodian or american, korean or american, vietnamese or americans, asian or americans, cambodian or americans, chinese or americans, filipino or americans, hmong or americans, japanese or americans, korean or americans, vietnamese or asian american or asian americans or asian indian american or asian indian americans or cambodian american or cambodian americans or chinese american or chinese americans or filipino american or filipino americans or hmong american or hmong americans or indian american, asian or japanese american or japanese americans or korean american or korean americans or vietnamese american or vietnamese americans).mp. | 115,813 |
| **19** | (native* or "native hawaiian" or "native-hawaiian" or samoan* or chamorro* or tonga* or fiji* or marshallese* or tahiti* or "pacific islander" or "pacific-islander").mp. | 32,376 |
| **20** | Oceanic Ancestry Group/ or (aborigine, australian or aborigines, australian or ancestry group, oceanic or ancestry groups, oceanic or australian aborigine or australian aborigines or australian race or oceanic ancestry or groups, oceanic ancestry or hawaiian, native or hawaiians, native or native hawaiian or native hawaiians or oceanic ancestry group or oceanic ancestry groups or pacific island american or pacific island americans or pacific islander american or pacific islander americans or race, australian or races, australian or races).mp. | 2,057 |
| **21** | (latin* or hispan* or arab* or arabic* or "middle-east*" or "Middle East*").mp. | 73,694 |
| **22** | Hispanic Americans/ or Mexican Americans/ or (american, hispanic or americans, cuban or americans, hispanic or americans, spanish or cuban american or cuban americans or hispanic or hispanic american or hispanic americans or hispanics or latina or latinas or latino or latinos or puerto rican or puerto ricans or spanish american or spanish americans).mp. or (american, mexican or americans, mexican or chicana or chicanas or mexican american or mexican americans).mp. | 57,435 |
| **23** | Amish/ or Arabs/ or Roma/ or Jews/ or (arab or arabs or bedouin or bedouins or palestinian or palestinians).mp. or (roma or romani).mp. or (jew or jews).mp. | 15,440 |
| **24** | or/4-23 | 694,355 |
| **25** | 1 AND 2 | 2,412 |
| **26** | 3 OR 25 | 2,427 |
| **27** | 24 AND 26 | 319 |

**Supplement Table 7:** *Search Terms and Number of Records for Open Grey*

| **Number** | **Open Grey** |  |
| --- | --- | --- |
|  | **Search Terms** | **Results** |
| **1** | (("clozapine prescription" or "clozapine prescribing" or "clozapine prescri*" or "prescription of clozapine" or "prescriptions of clozapine" or "prescri* of clozapine" or "prescribing of clozapine" or "use of clozapine" or "uses of clozapine" or "usage of clozapine" or "usages of clozapine" or "clozapine use" or "clozapine-use" or "clozapine uses" or "clozapine usage" or "clozapine usages" or "utilisation of clozapine" or "utilization of clozapine" or "utilisations of clozapine" or "utilizations of clozapine" or "utili* of clozapine" or "clozapine utili*" or "clozapine utilisation" or "clozapine utilization" or "clozapine utilisations" or "clozapine utilizations" or "utilising clozapine" or "utilizing clozapine" or "utility of clozapine" or "clozapine initiation" or "clozapine initia*" or "initiation of clozapine" or "initia* of clozapine" or "initiating clozapine") | 0 |
| **2** | (clozapine or zaponex or denzapin* or clozaril or leponex) | 0 |
| **3** | (prescription or prescribing or prescri* or "prescription of" or "prescriptions of" or "prescri* of" or "prescribing of" or "use of" or "uses of" or "usage of" or "usages of" or uses or usage O usages or "utilisation of" or "utilization of" or "utilisations of" or "utilizations of" or "utili* of" or utilisation or utilization or utilisations or utilizations or utilising or utilizing or utility or "utility of" or initiation or intia* or "initiations of" or initiating) | 0 |
| **4** | ((((((refugee* OR immigrant* OR (asyl* adj1 seek*) OR foreign* OR ethnic* OR "ethnic groups" OR "ethnic-groups" OR "minority groups" OR "population groups" OR minorit* OR race OR racial* OR multiethnic* OR multi-ethnic* OR nationalit* OR nation* OR "foreign-national" or "foreign national" OR "non-white" OR "non white" OR "non-national" OR "non national" OR tribe*)) OR ((indig* OR aborig* OR "first people*" OR "first australian*" OR "first-australian*" OR native* OR "native american*" OR "native-american*" OR inuit* OR maori OR niuean*))) OR ((black OR "black british" OR "black-british" OR "african american" OR "african-american" OR african* OR caribbean* OR afro-caribbean* OR afrocaribbean* OR afro* OR "BAME" OR "BME"))) OR ((asian OR "asian british" OR "asian-british" OR "asian american*" OR "asian-american*" OR indian* OR pakistani* OR bangladeshi* OR chinese or korean OR japanese OR filipin* OR viet* "asian-indian" OR "asian indian"))) OR ((native* OR "native hawaiian" OR "native-hawaiian" OR samoan* OR chamorro* OR tonga* OR fiji* OR marshallese* OR tahiti* OR "pacific islander" OR "pacific-islander"))) OR ((latin* or hispan* or arab* or arabic* or "middle-east*" or "Middle East*")) | 98,154 |
| **5** | 2 AND 3 | 0 |
| **6** | 1 OR 5 | 0 |
| **7** | 6 AND 4 | 0 |

**Supplement Table 8:** *Headings for Data Extraction Form*

| Author |  |
| --- | --- |
| Year |  |
| Title |  |
| Country |  |
| Study Design |  |
| Source of Sample |  |
| Years/Observation Period |  |
| Inclusion Criteria (for sample) |  |
| Definition of TRS used |  |
| Missing Data |  |
| Diagnosis |  |
| N (total of sample) |  |
| Ethnic Groups |  |
| Results (descriptive) - % of clozapine prescription for each ethnic group |  |
| Results (inferential) – results of association tests |  |
| Type of Analysis Conducted |  |
| Confounders adjusted for/controlled for |  |
| Confounders not controlled for (bias) |  |
| Limitations |  |
| Ways any bias was addressed |  |
| Quality Assessment Score^a^ |  |

*Notes.* Template of data extraction form, data extraction was carried out on an Excel spreadsheet with these headings.

^a^Only information that were relevant to this review question were assessed using this quality assessment tool, to assess the quality of information of each included study within the context of this review. Thereby, the quality assessment score awarded is not reflective of the study quality relevant to the original study aim.

**Supplementary Table 9:** *Modified version of the Johanna Briggs Institute Critical Appraisal Checklist of Cross-Sectional Studies (adapted from Joanna Briggs Institute, 2020)*

| **Item Number** | **Question** | **Response** |
| --- | --- | --- |
| **1** | Were the criteria for inclusion in the sample clearly defined? | Y N U |
| **2** | Were the study subjects and the setting described in detail? | Y N U |
| **3** | Were objective, standard criteria used for the measurement of the schizophrenia diagnosis (i.e., DSM or ICD or clinicians’ decision)? | Y N U |
| **4** | Was the prescribing of clozapine measured in a valid and reliable way? | Y N U |
| **5** | Was the sample composed only of patients eligible to receive clozapine (i.e., only including TRS samples)? | Y N U |
| **6** | Was statistical analysis used? | Y N U |
| **7** | Were analytic strategies to deal with demographic confounding factors (e.g., gender, age) employed (e.g., multivariable, univariable analyses (x^2^)? | Y N U |
| **8** | Were analytic strategies to deal with clinical confounding factors (e.g., symptoms, comorbidities, neutropenia and service use) employed (e.g., multivariable analyses, using propensity scores for confounding by indication, univariable analyses (x^2^))? | Y N U |
| **9** | Was the sample frame appropriate and the study participants recruited in an appropriate way? Were people from different ethnicities recruited from same sources (e.g., location, clinics)? Was recruitment was done with outpatients and inpatients information? Did recruitment include convenience samples? Did they analyse all records, or a non-random sample? Was the sample representative of that geographical area? | Y N U |
| **10** | Was the sample size adequate? | Y N U |
| **11** | Was the response rate adequate, and if not, was the low response rate/high drop-out rate managed appropriately? Did a high proportion of people eligible for inclusion (according to sampling frame) end up in the study? Was there missing data? | Y N U |

*Notes.* One point was awarded for every ‘Yes’ on each item. Total score calculated as the sum of all awarded points. Total score was used to assign each included study into one of three categories of quality of information: High (10-11 points), Medium (8-9 points), Low (0-7 points). Abbreviations: Y, Yes; N, No; U, Unclear.

**Supplementary** **Table 10:** *Quality Assessment Scores of Included Studies*

| **Citation** | **Q1** | **Q2** | **Q3** | **Q4** | **Q5** | **Q6** | **Q7** | **Q8** | **Q9** | **Q10** | **Q11** | **Total Score** | **Category** |
| --- | --- | --- | --- | --- | --- | --- | --- | --- | --- | --- | --- | --- | --- |
| Das-Munshi et al., (2018) | Y | Y | Y | Y | Y | Y | Y | Y | N | Y | Y | 10/11 points | High |
| Kuno and Rothbard, (2002) | Y | Y | Y | Y | N | Y | Y | Y | Y | Y | Y | 10/11 points | High |
| Manuel et al., (2012) | Y | Y | Y | Y | N | Y | Y | Y | Y | Y | Y | 10/11 points | High |
| Stroup et al., (2014) | Y | Y | Y | Y | Y | Y | Y | Y | Y | U | Y | 10/11 points | High |
| Cho et al., (2019) | Y | Y | Y | Y | Y | Y | N | N | Y | Y | Y | 9/11 points | Medium |
| Copeland et al., (2003) | Y | Y | Y | Y | N | Y | Y | N | Y | Y | Y | 9/11 points | Medium |
| Rothbard et al., (2003) | Y | Y | Y | Y | N | Y | N | N | Y | Y | Y | 8/11 points | Medium |
| Wheeler et al., (2008) | Y | Y | Y | Y | N | Y | Y | N | Y | Y | Y | 9/11 points | Medium |
| Wheeler, (2008) | Y | Y | Y | Y | N | Y | N | N | Y | Y | Y | 8/11 points | Medium |
| Beck et al., (2019) | Y | Y | Y | Y | Y | Y | N | N | N | N | Y | 7/11 points | Low |
| de Almeida et al., (2020) | Y | Y | U | N | N | N | N | N | Y | N | U | 3/11 points | Low |
| Dey , et al., (2016) | Y | N | Y | U | N | Y | N | N | N | N | Y | 4/11 points | Low |
| Govind et al., (2020) | Y | Y | Y | Y | N | N | N | N | Y | Y | Y | 7/11 points | Low |
| Horvitz-Lennon et al., (2013) | Y | Y | Y | Y | N | N | N | N | Y | Y | Y | 7/11 points | Low |
| Horvitz-Lennon et al., (2014) | Y | Y | Y | Y | N | N | N | N | N | U | U | 4/11 points | Low |
| Kelly et al., (2006) | Y | Y | Y | Y | U | Y | N | N | U | Y | Y | 7/11 points | Low |
| Kelly et al., (2010) | Y | Y | Y | Y | N | Y | N | N | N | Y | N | 6/11 points | Low |
| Kesserwani et al., (2019) | Y | Y | Y | Y | N | U | N | N | Y | Y | Y | 7/11 points | Low |
| Kreyenbuhl et al., (2003) | Y | U | Y | Y | N | Y | N | N | N | N | N | 4/11 points | Low |
| Mallinger et al., (2006) | Y | Y | Y | Y | N | Y | Y | N | Y | N | N | 7/11 points | Low |
| Mark et al., (2003) | Y | Y | Y | Y | N | N | N | N | N | Y | Y | 6/11 points | Low |
| Ren et al., (2002) | Y | Y | Y | Y | N | Y | N | N | N | Y | N | 6/11 points | Low |
| Stokes et al., (2020) | Y | Y | Y | Y | Y | Y | N | N | U | Y | N | 7/11 points | Low |
| Velligan et al., (2015) | Y | Y | Y | Y | N | N | N | N | Y | Y | N | 6/11 points | Low |

*Notes.* Quality assessment of each included study evaluated using a modified version of the Johanna Briggs Institute Critical Appraisal Checklist of Cross-Sectional Studies. Study quality was only assessed with regards to information relevant to this review, so the quality assessment score awarded does not necessarily reflect the study quality regarding its primary aim.1 point awarded for every ‘Yes’ on each item. Total score calculated as the sum of all awarded points. Total score was used to assign each included study into a category of quality of information: High (10-11 points), Medium (8-9 points), Low (0-7 points). Abbreviations: Y, Yes; N, No; U, Unclear.
